# Supplementary material for: Ecological niche modelling does not support climatically-driven dinosaur diversity decline before the Cretaceous/Paleogene mass extinction
Source: Nat Commun. 2019 Mar 6;10:1091. doi: 10.1038/s41467-019-08997-2 (PMC6403247; doi:10.1038/s41467-019-08997-2)
Supplement: Supplementary file 1 — Supplementary Information [file 41467_2019_8997_MOESM1_ESM.pdf]

SUPPLEMENTARY INFORMATION

Ecological niche modelling does not support climatically-driven dinosaur diversity decline before the Cretaceous/Paleogene mass extinction

Chiarenza et al.

Supplementary Note 1: Environmental predictors used for ENM analysis

Variables were chosen based on which parameters are thought to have a direct physiological influence on the organism. Although we cannot directly determine the thermophysiology or other autoecological features of extinct members of Dinosauria, coarse phylogenetic inferences can be suggested based on their most closely related living organisms, sauropsids[1], particularly crown group archosaurs (crocodiles[2] + birds[3]). Temperature variables have been commonly used in phylogenetic niche conservation studies across clades[4], as they represent some degree of realism with respect to how maximum and minimum temperature values vary and affect environmental conditioning across landscapes[5]. The use of these variables with a 'broad' ecologically important determining effect reduces assumptions on the thermophysiology of these dinosaur clades, which is still debated (e.g. Grady et al.[6]). Furthermore, the precipitation metric might have secondarily influenced the distribution of dinosaur communities by affecting plants' distribution patterns, with a bottom–up effect in structuring the consumers[7]. Indirect predictors like topography were not used in this study as we are interested in macroscale patterns, and topography is usually indirectly associated with generating heterogeneity in microclimate that can provide microscale variability in an organism's environment[3]. In addition, given the large size of the animals investigated in this study, we use the assumption than microclimatic patterns had a minor effect on their spatial distribution at continental scale. Figures, dendrograms, and matrices of correlation analyses are plotted in R version 3.4.4. (R core team 2017).

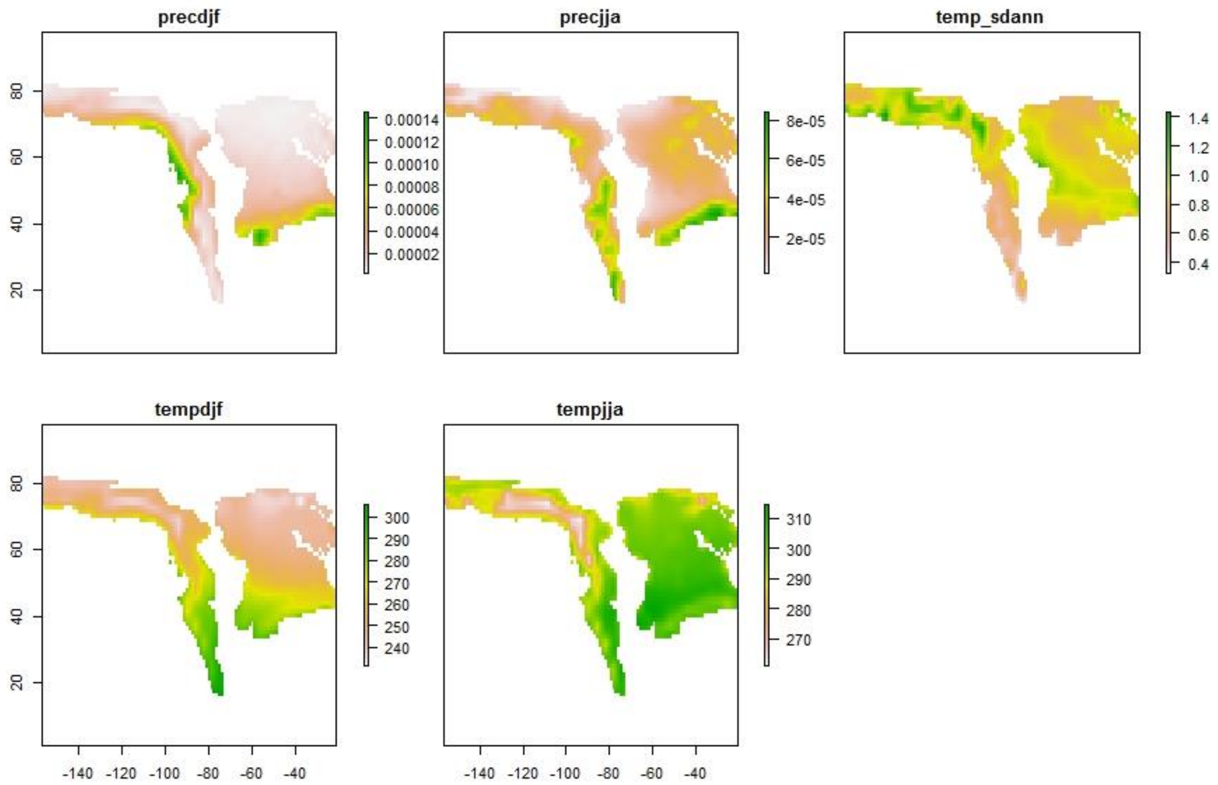

Supplementary Figure 1. Environmental predictors used for Campanian ENM analysis. Abbreviations: precipitation in the driest quarter (precdjg); precipitation in the wettest quarter (precjja); annual temperature standard deviation (temp_sdann); temperature in the coldest quarter (tempdjf) and temperature in the warmest quarter (tempjja). All plots and maps made by AAC with data from AAC and coauthors and reported herein.

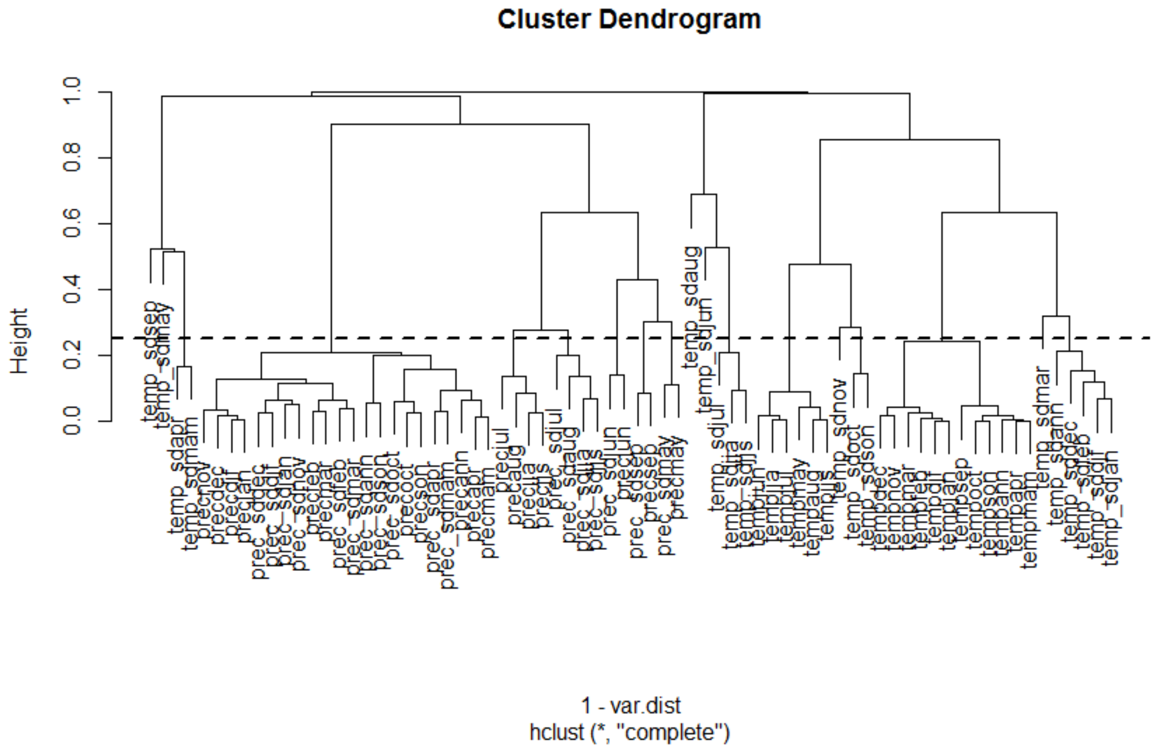

Supplementary Figure 2. Hierarchical cluster dendrogram of correlation analyses between Campanian environmental predictors. Pearson's correlation test to explore co-linearity between variables, retaining only the ones showing a Pearson’s correlation coefficient of less than 0.7 (dashed line) in order to minimise multicolinearity between variables. Abbreviations: precipitation (prec); temperature (temp); standard deviation (sd); annual (ann). January (Jan), February (Feb), March (Mar), April (Apr), May (May), June (Jun), July (Jul), August (Aug), September (Sep), October (Oct), November (Nov), December (Dec).

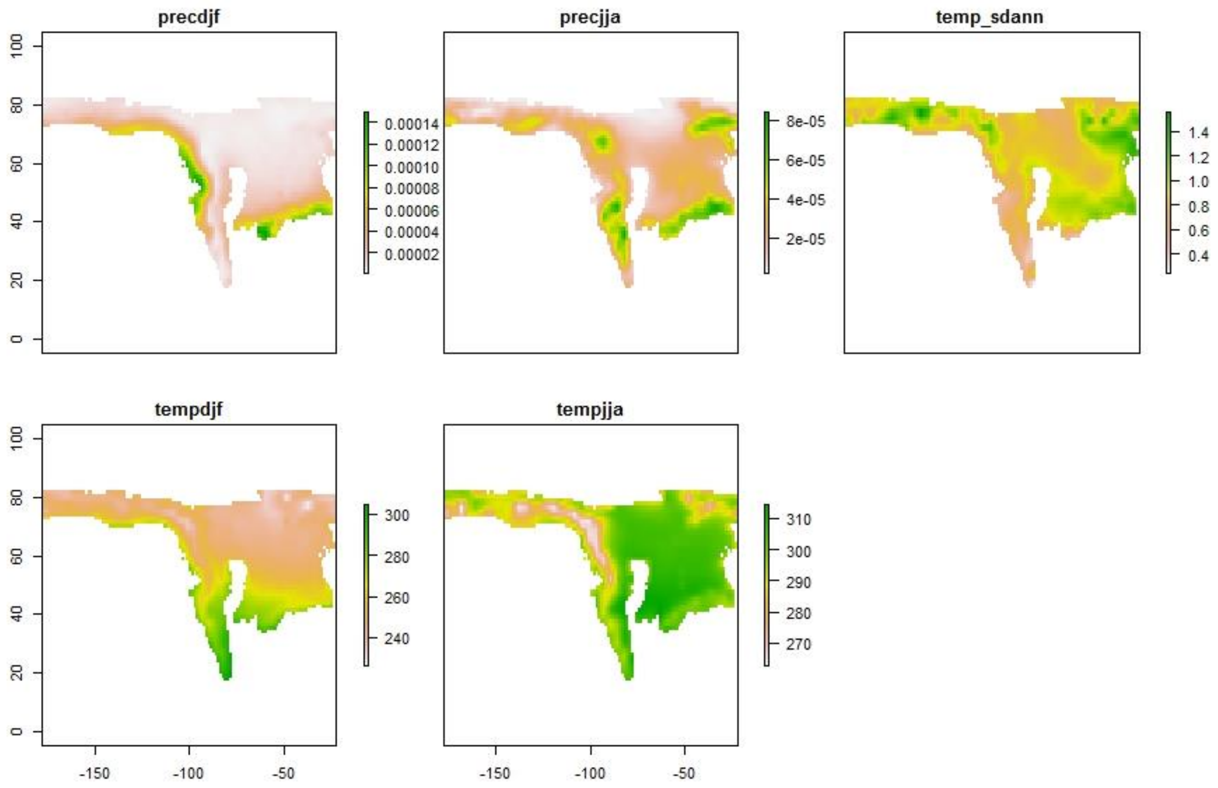

Supplementary Figure 3. Environmental predictors used for Maastrichtian ENM analysis. Abbreviations: precipitation in the driest quarter (precdjg); precipitation in the wettest quarter (precjja); annual temperature standard deviation (temp_sdann); temperature in the coldest quarter (tempdjf) and temperature in the warmest quarter (tempjja). All plots and maps made by AAC with data from AAC and coauthors and reported herein.

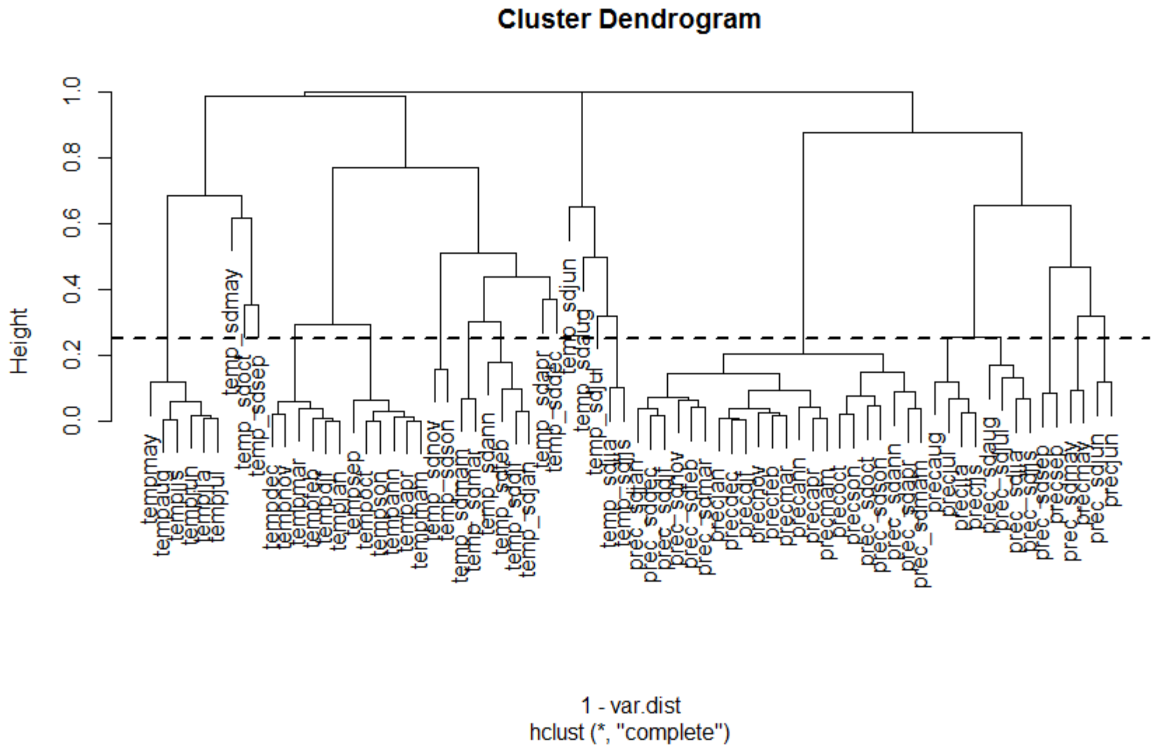

Supplementary Figure 4. Hierarchical cluster dendrogram of correlation analyses between Maastrichtian environmental predictors. Pearson's correlation test to explore co-linearity between variables, retaining only the ones showing a correlation coefficient of less than 0.7 (dashed line) in order to minimise multicolinearity between variables. Abbreviations: precipitation (prec); temperature (temp); standard deviation (sd); annual (ann). January (Jan), February (Feb), March (Mar), April (Apr), May (May), June (Jun), July (Jul), August (Aug), September (Sep), October (Oct), November (Nov), December (Dec). All plots and maps made by AAC with data from AAC and coauthors and reported herein.

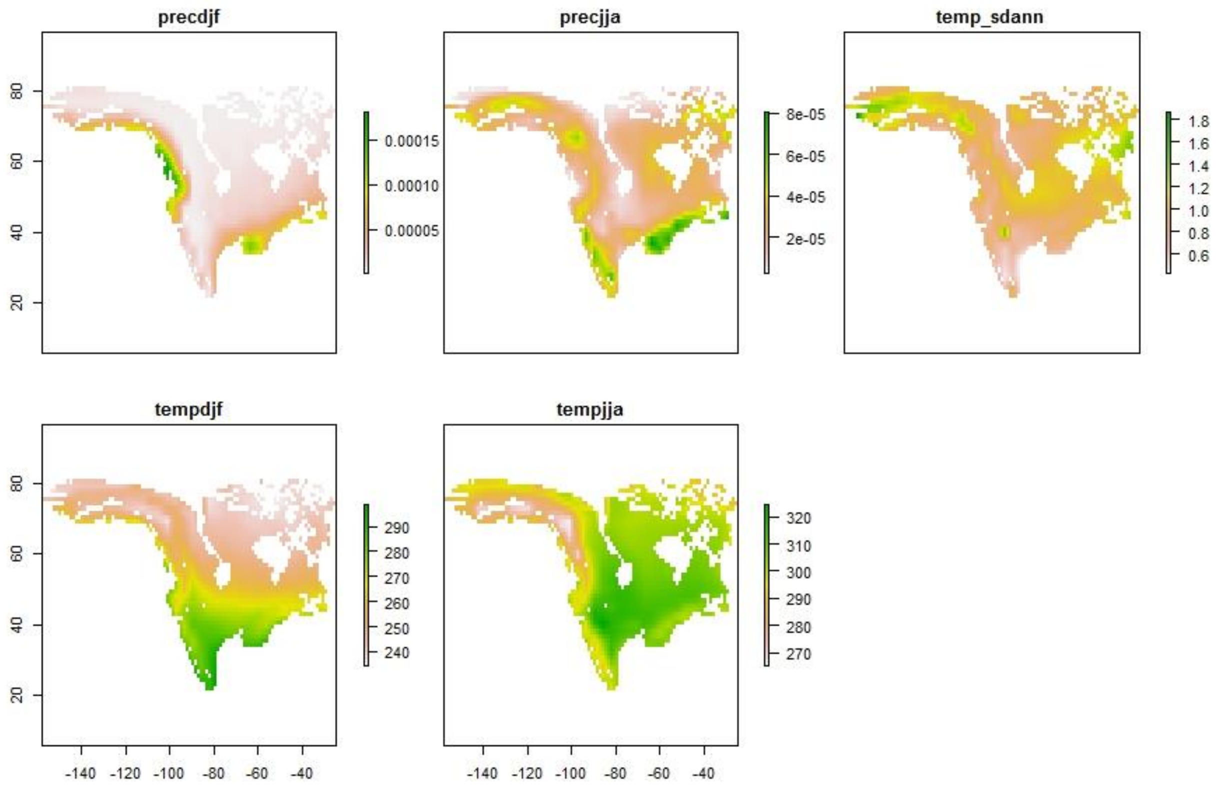

Supplementary Figure 5. Environmental predictors used for Danian ENM projections. Abbreviations: precipitation in the driest quarter (precdjg); precipitation in the wettest quarter (precjja); annual temperature standard deviation (temp_sdann); temperature in the coldest quarter (tempdjf) and temperature in the warmest quarter (tempjja). All plots and maps made by AAC with data from AAC and coauthors and reported herein.

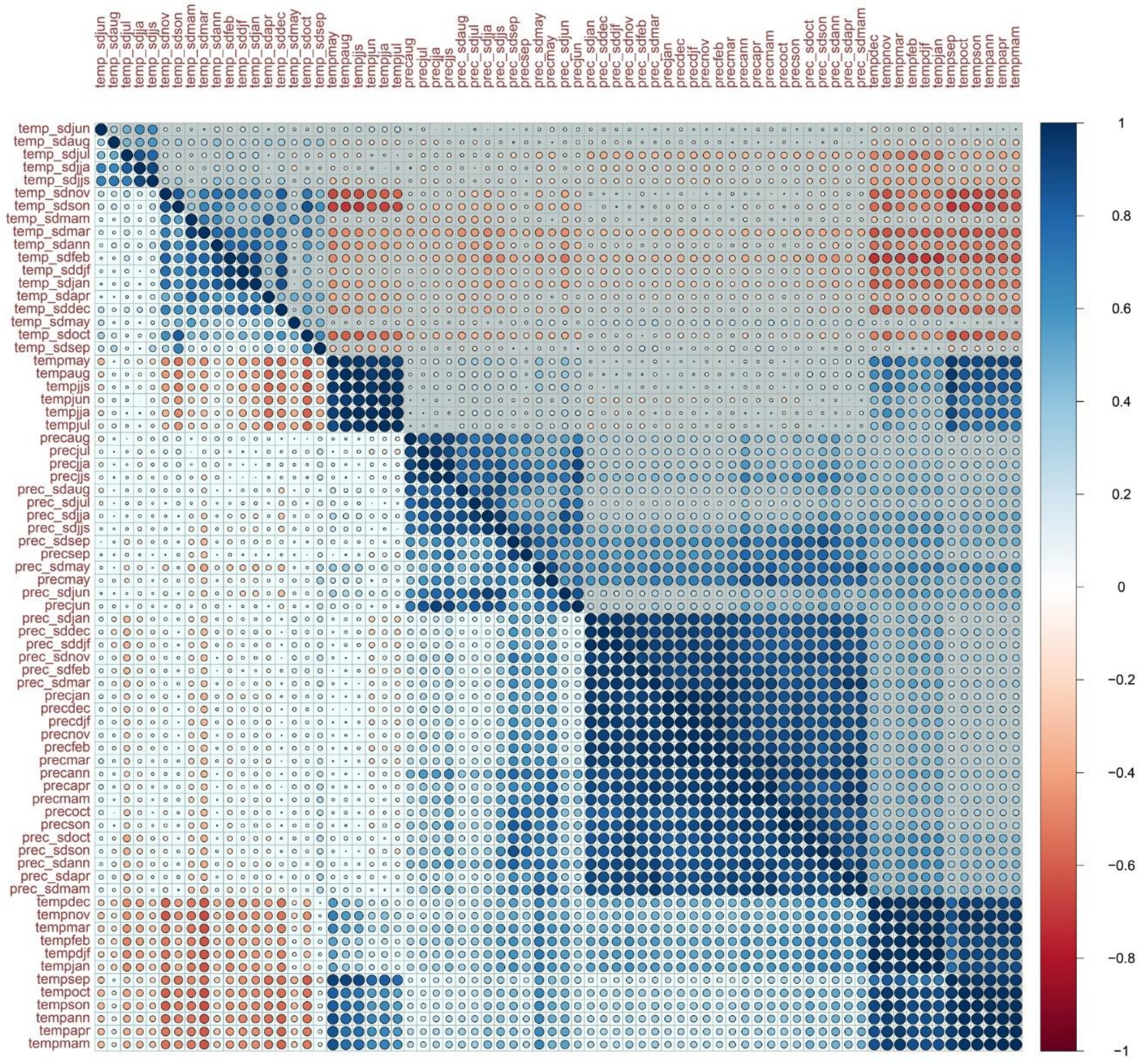

Supplementary Figure 6. Correlation matrix between Campanian and Maastrichtian environmental predictors. Blue indicates high positive correlation while red indicates high negative correlation. Neutral white colour indicates no correlation. Abbreviations: precipitation (prec); temperature (temp); standard deviation (sd); annual (ann). January (Jan), February (Feb), March (Mar), April (Apr), May (May), June (Jun), July (Jul), August (Aug), September (Sep), October (Oct), November (Nov), December (Dec).

Supplementary Note 2: Hotspot analysis with Kernel density on latest Cretaceous fossil occurrences

Hotspot analysis highlights spatial association of densely packed localities (red) that contribute to biasing our interpretation of palaeodiversity trends (see also Close et al.[8], and Plotnick[9]). Grey areas represent landmasses, with solid line representing sea level lowstand. Palaeo-rotated country boundaries are superimposed onto palaeogeography. The star present in some figures represents Dinosaur Provincial Park, one of the richest dinosaur bearing localities in the world.

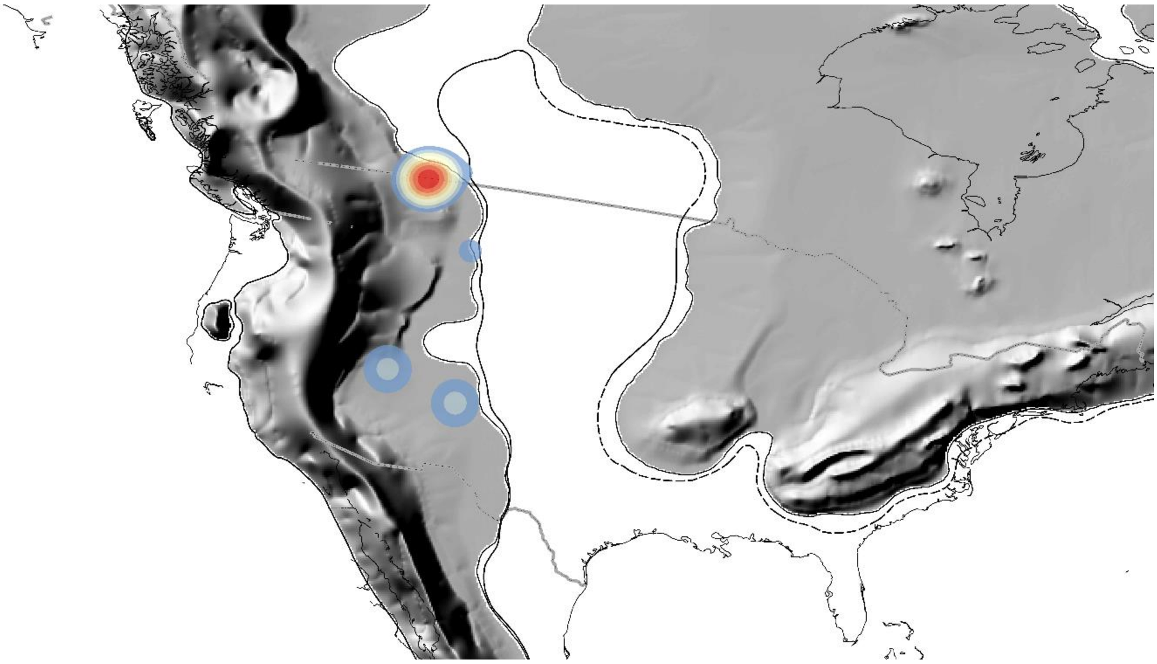

Supplementary Figure 7. Early Campanian Ceratopsidae. Colours indicates low density (blue) and high density (red) of fossil localities. All plots and maps made by AAC with data from AAC and coauthors and reported herein.

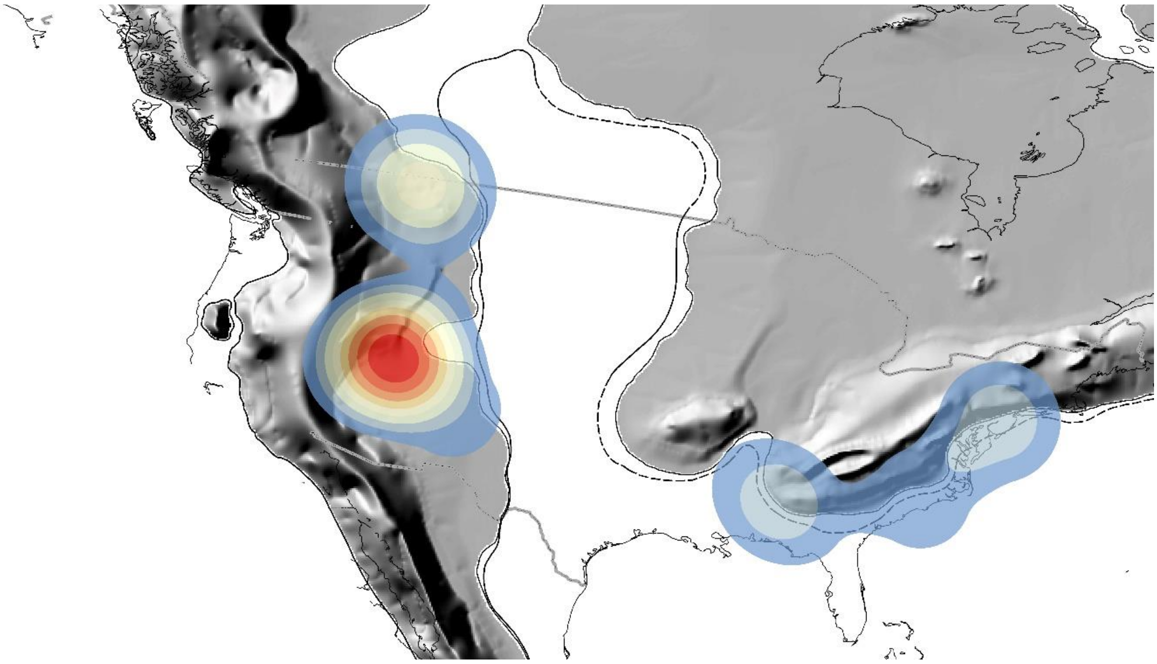

Supplementary Figure 8. Early Campanian Hadrosauridae. Colours indicates low density (blue) and high density (red) of fossil localities. All plots and maps made by AAC with data from AAC and coauthors and reported herein.

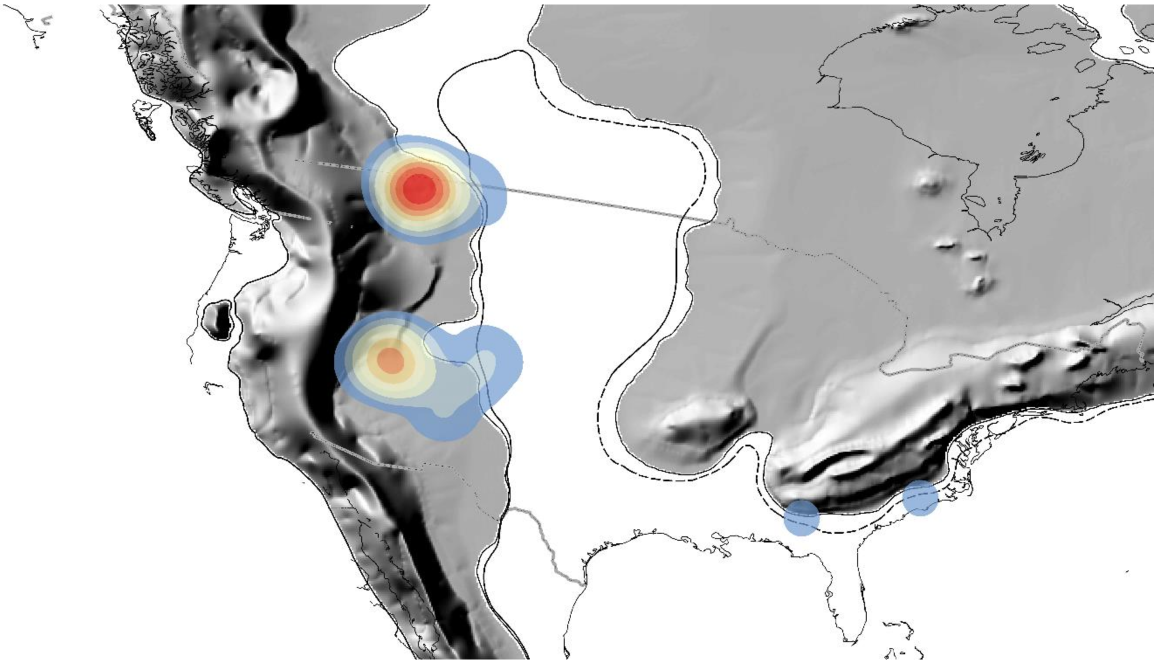

Supplementary Figure 9. Early Campanian Tyrannosauridae. Colours indicates low density (blue) and high density (red) of fossil localities. All plots and maps made by AAC with data from AAC and coauthors and reported herein.

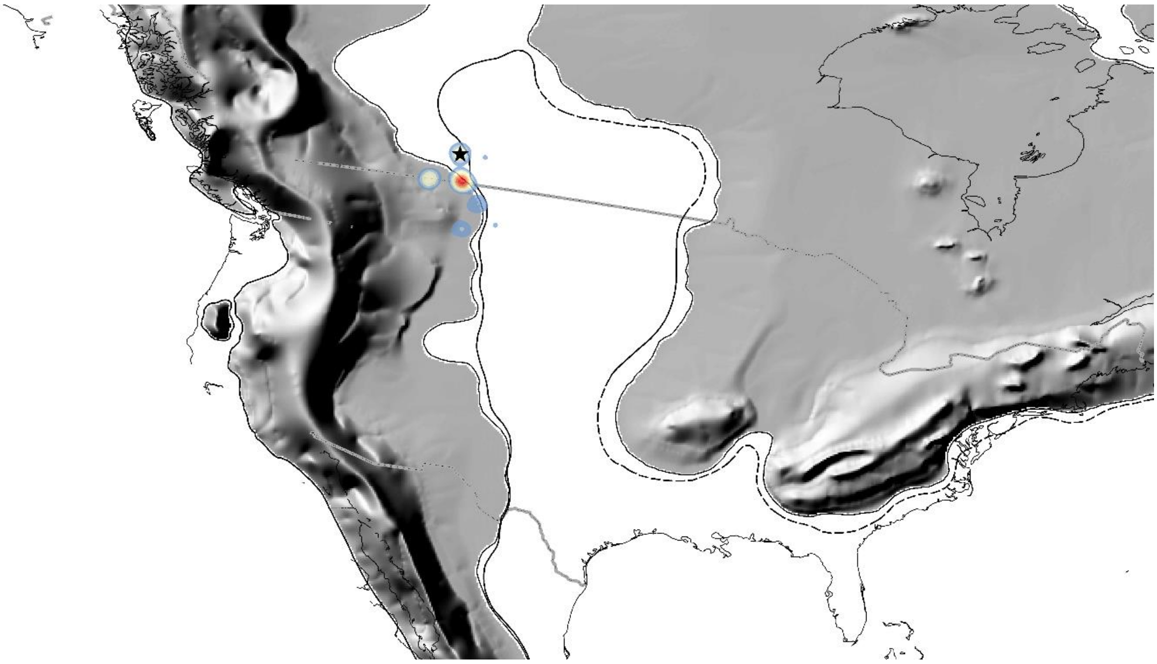

Supplementary Figure 10. Middle Campanian Ceratopsidae. Colours indicates low density (blue) and high density (red) of fossil localities. All plots and maps made by AAC with data from AAC and coauthors and reported herein.

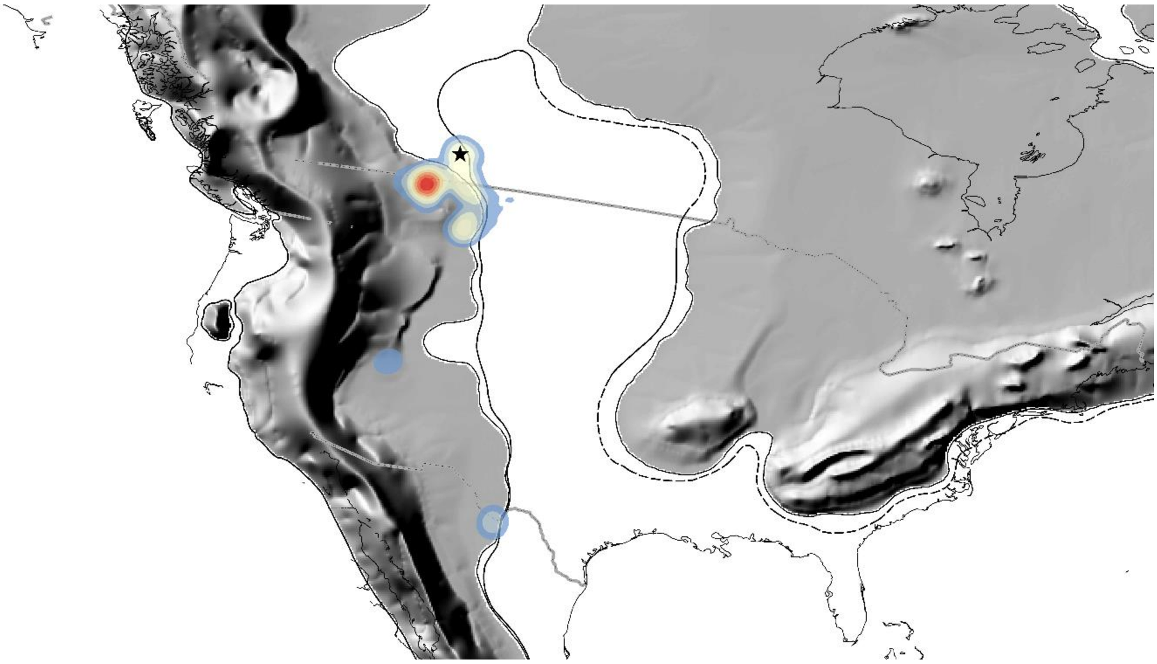

Supplementary Figure 11. Middle Campanian Hadrosauridae. Colours indicates low density (blue) and high density (red) of fossil localities. All plots and maps made by AAC with data from AAC and coauthors and reported herein.

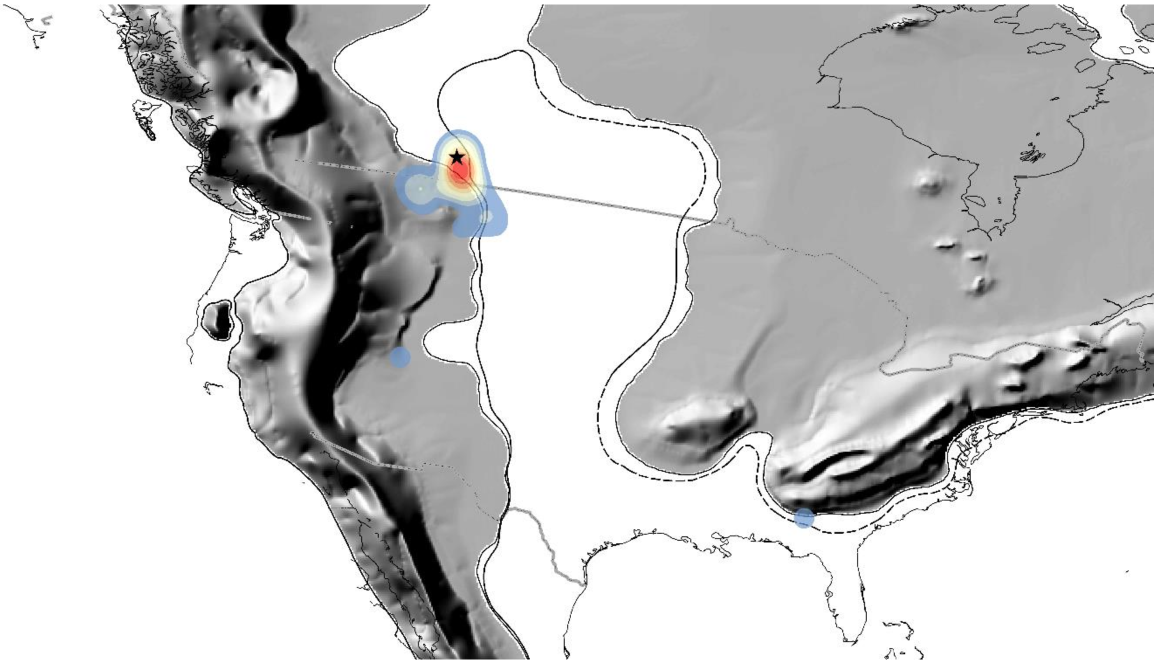

Supplementary Figure 12. Middle Campanian Tyrannosauridae. Colours indicates low density (blue) and high density (red) of fossil localities. All plots and maps made by AAC with data from AAC and coauthors and reported herein.

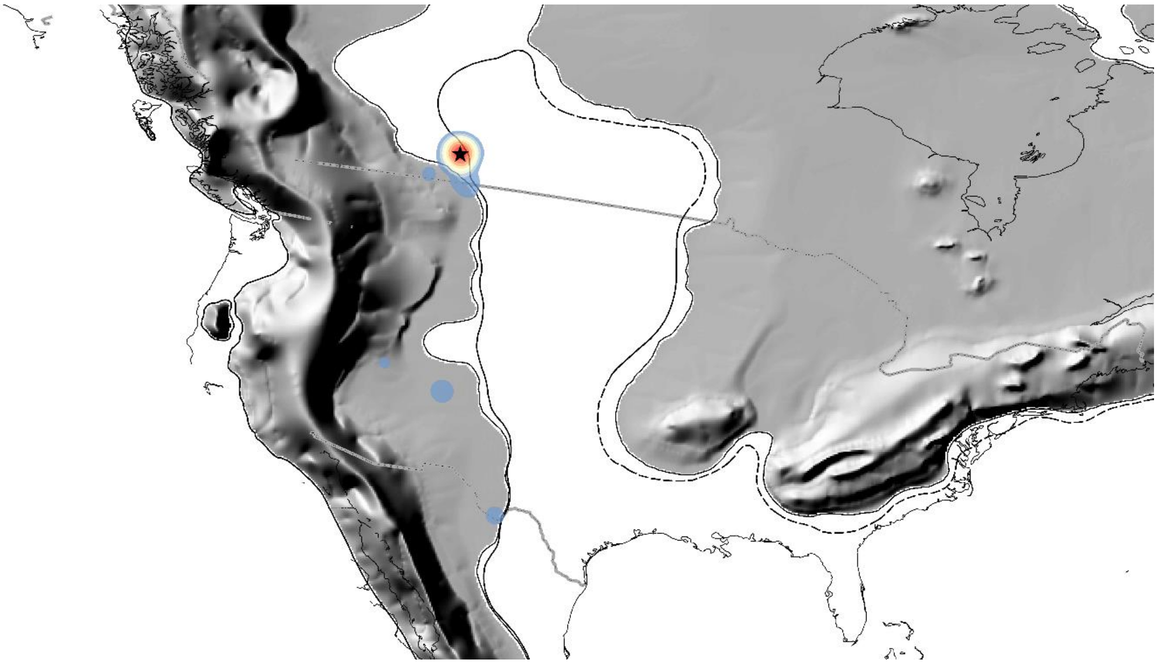

Supplementary Figure 13. Late Campanian Ceratopsidae. Colours indicates low density (blue) and high density (red) of fossil localities. All plots and maps made by AAC with data from AAC and coauthors and reported herein.

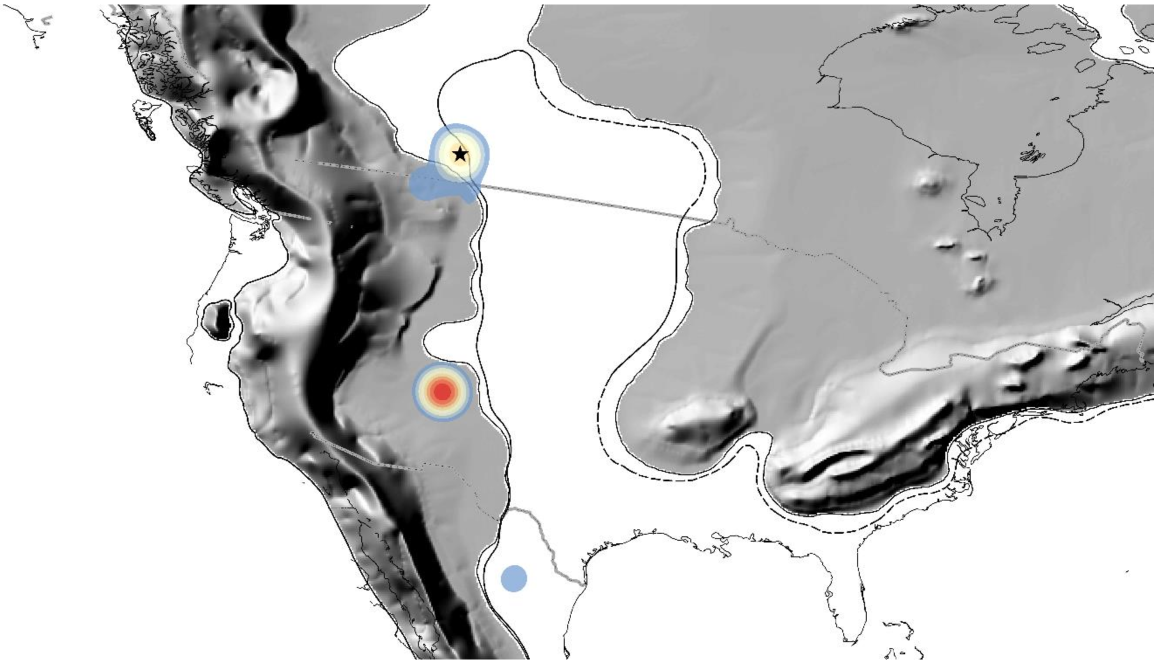

Supplementary Figure 14. Late Campanian Hadrosauridae. Colours indicates low density (blue) and high density (red) of fossil localities. All plots and maps made by AAC with data from AAC and coauthors and reported herein.

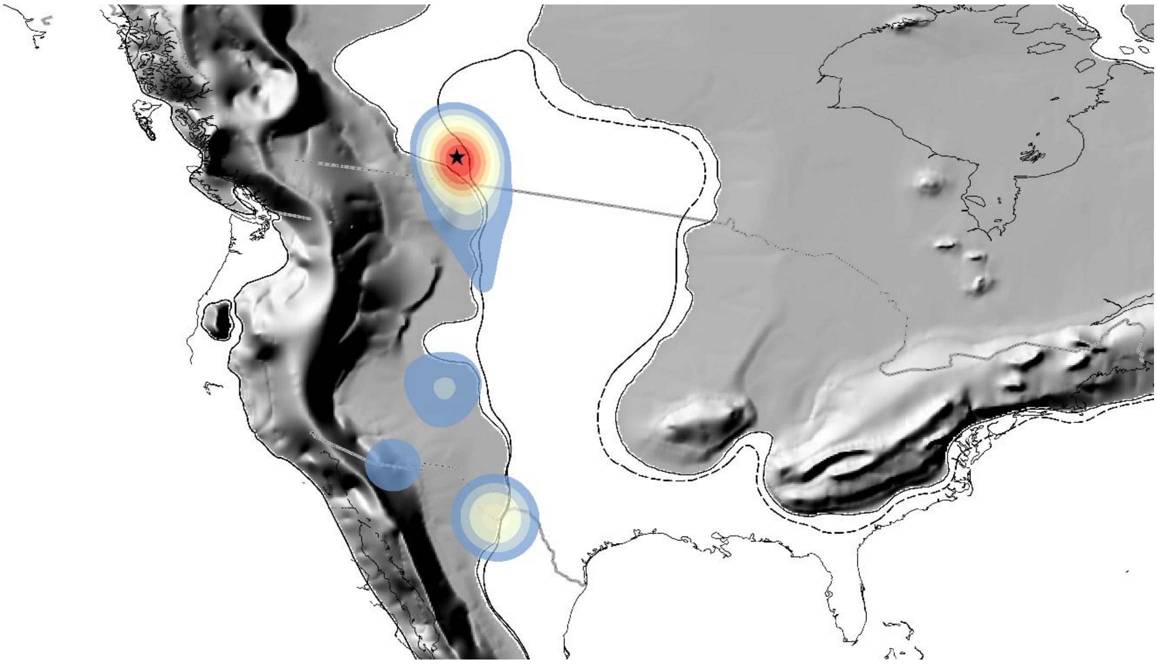

Supplementary Figure 15. Late Campanian Tyrannosauridae. Colours indicates low density (blue) and high density (red) of fossil localities. All plots and maps made by AAC with data from AAC and coauthors and reported herein.

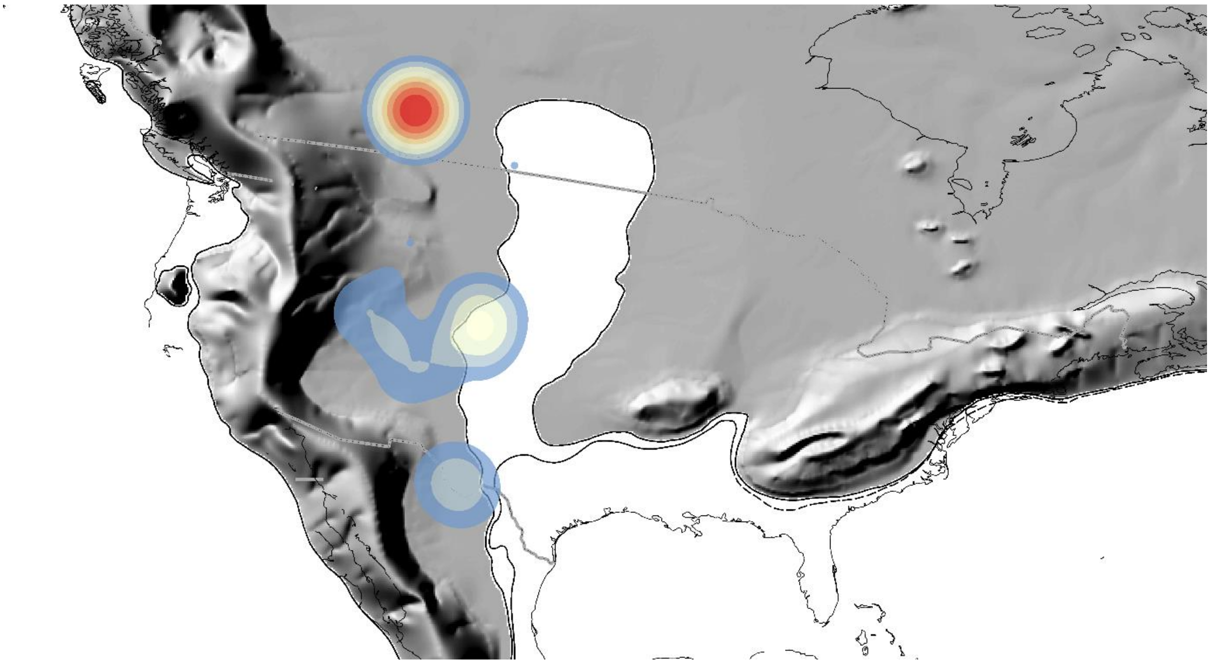

Supplementary Figure 16. Early Maastrichtian Ceratopsidae. Colours indicates low density (blue) and high density (red) of fossil localities. All plots and maps made by AAC with data from AAC and coauthors and reported herein.

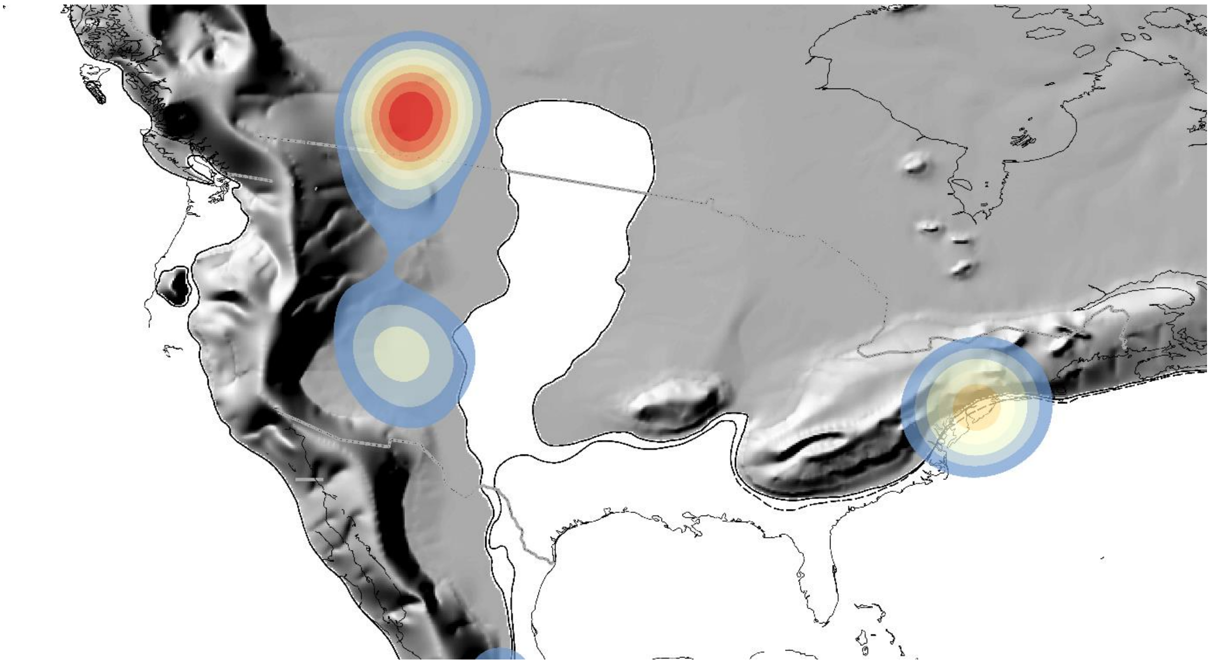

Supplementary Figure 17. Early Maastrichtian Hadrosauridae. Colours indicates low density (blue) and high density (red) of fossil localities. All plots and maps made by AAC with data from AAC and coauthors and reported herein.

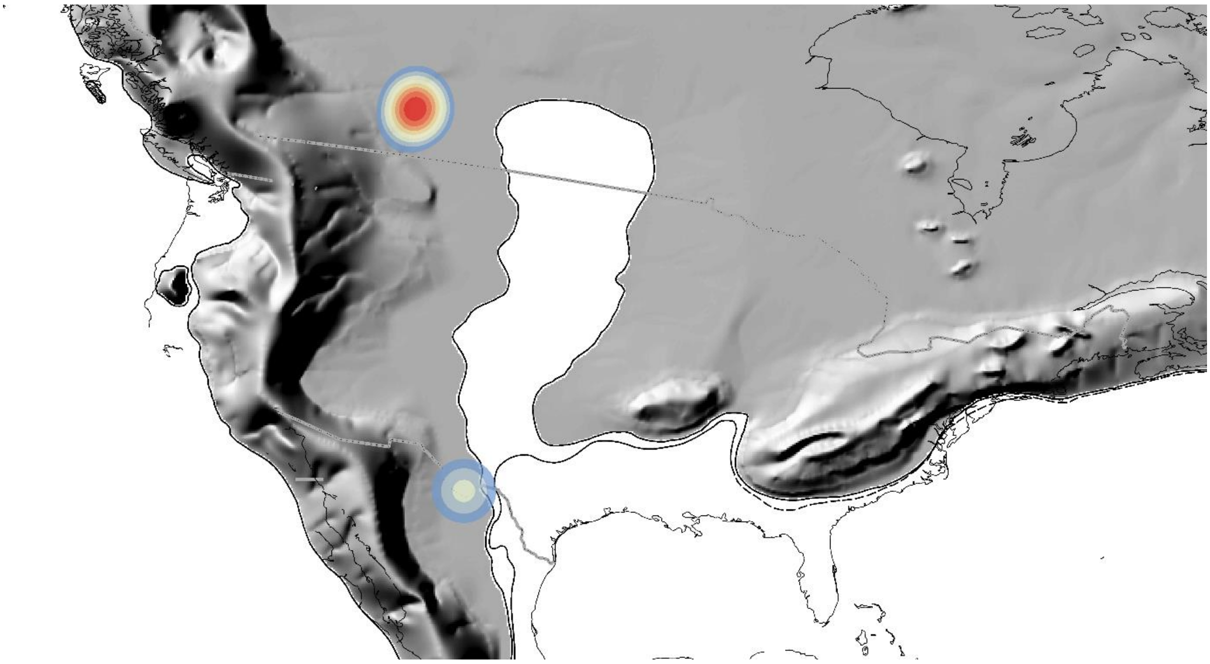

Supplementary Figure 18. Early Maastrichtian Tyrannosauridae. Colours indicates low density (blue) and high density (red) of fossil localities. All plots and maps made by AAC with data from AAC and coauthors and reported herein.

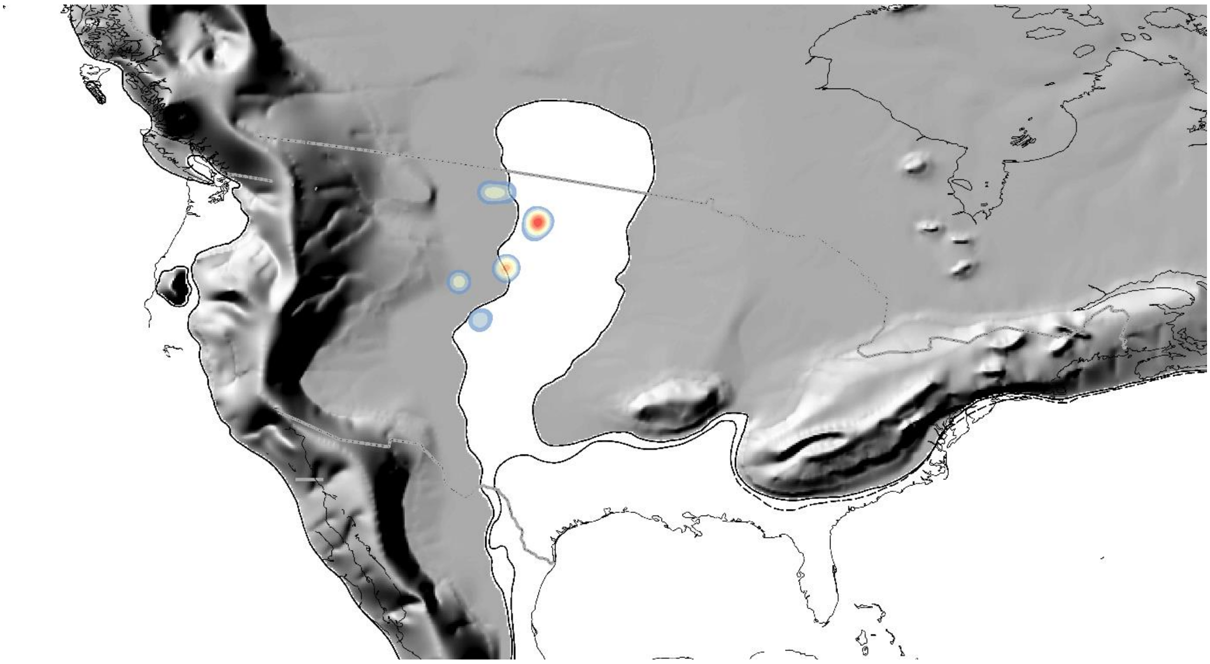

Supplementary Figure 19. Late Maastrichtian Ceratopsidae. Colours indicates low density (blue) and high density (red) of fossil localities. All plots and maps made by AAC with data from AAC and coauthors and reported herein.

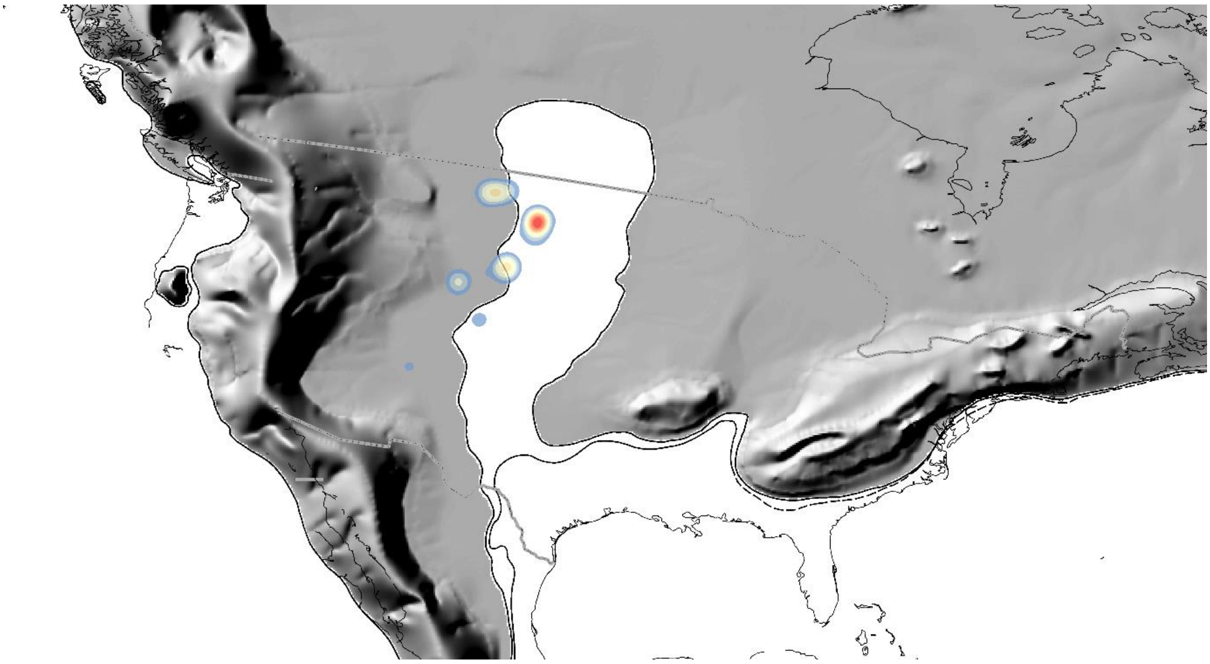

Supplementary Figure 20. Late Maastrichtian Hadrosauridae. Colours indicates low density (blue) and high density (red) of fossil localities. All plots and maps made by AAC with data from AAC and coauthors and reported herein.

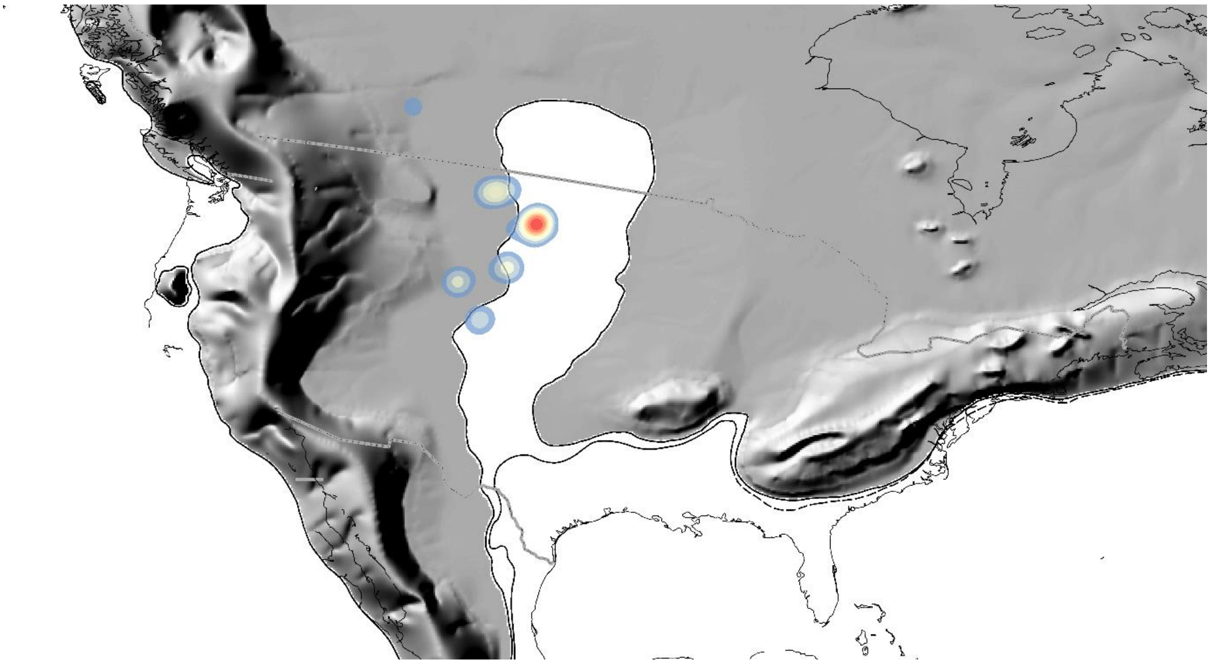

Supplementary Figure 21. Late Maastrichtian Tyrannosauridae. Colours indicates low density (blue) and high density (red) of fossil localities. All plots and maps made by AAC with data from AAC and coauthors and reported herein.

Supplementary Table 1: Quantification of ecological niche models for the outcrop areas through the latest Cretaceous of North American. These values are used for the plots in Figure 4a. Numeric values in columns are in square km. Numbers in row titles (0.2, 0.45 and 0.7) are the thresholds used to quantify areas of habitat suitability.

Taxon	Early Campanian (km²)	Middle Campanian (km²)	Late Campanian (km²)	Early Maastrichtian (km²)	Late Maastrichtian (km²)
Ceratopsidae (0.2)	1223807	1289321	1305203	1452370	1448372
Hadrosauridae (0.2)	1206777	1274267	1215866	1670158	1357782
Tyrannosauridae (0.2)	1215866	1226617	1309173	1670158	1533309
Ceratopsidae (0.45)	1015789	625593.4	486691.7	311271.2	557511.2
Hadrosauridae (0.45)	842865.2	470913.9	566646.3	366450.8	545141.2
Tyrannosauridae (0.45)	917858.2	939833.3	781207.5	278738.1	622449.5
Ceratopsidae (0.7)	51871.53	186932	173520.8	55631.7	62347.73
Hadrosauridae (0.7)	297776.1	303917.7	297776.1	44528.45	86139.12
Tyrannosauridae (0.7)	296105.2	356899.4	253081.63	146686.3	90626.54

Supplementary Table 2: Quantification of ecological niche models for the whole latest Cretaceous North American palaeocontinent. These values are used for the plots in Figure 4b. Numeric values in columns are in square km. Numbers in row titles (0.2, 0.45 and 0.7) are the thresholds used to quantify areas of habitat suitability.

Taxon	Early Campanian (km²)	Middle Campanian (km²)	Late Campanian (km²)	Early Maastrichtian (km²)	Late Maastrichtian (km²)	Danian (km²)
Ceratopsidae (0.2)	10228907	15297770	14830387	15149188	14419877	11037503
Hadrosauridae (0.2)	8727389	14821112	11872570	21565459	12854895	11170601
Tyrannosauridae (0.2)	9214387	15013157	11872570	21565459	14489314	12486043
Ceratopsidae (0.45)	5058121	8643496	7994152	6247428	9937493	5086531
Hadrosauridae (0.45)	2798219	4672628	2192283	3303320	6489877	2932100
Tyrannosauridae (0.45)	2608187	2490453	4605138	1152635.9	3180084	3650077
Ceratopsidae (0.7)	2529390	5956264	4436891	3372027	5564646	2913958
Hadrosauridae (0.7)	1644602	1838327	2010481	759021.1	2815575	1408386
Tyrannosauridae (0.7)	346892.9	1464132	1816274	887117.2	706474.3	1064206

Supplementary References

1. Peterson, A. T., Ball, L. G. and Cohoon, K. P. Predicting distributions of Mexican birds using ecological niche modelling methods. *Ibis,* **144**, E27–E32 (2002).
2. Peterson, A. T. Predicting species' geographic distributions based on ecological niche modeling. *Condor*, **103**, 599–605 (2001).
3. Guisan, A. and Hofer, U. Predicting reptile distributions at the mesoscale: relation to climate and topography. *J. of Biogeog.* **30**, 1233–1243 (2003).
4. Peterson, A. T. Ecological niche conservatism: a time–structured review of evidence. *J. Biogeogr.* **38**, 817–827 (2011).
5. Saupe, E. E. et al. Reconstructing ecological niche evolution when niches are incompletely characterized. *Syst. Biol.* **67**, 428–438 (2018).
6. Grady, J. M., Enquist, B. J., Dettweiler–Robinson, E., Wright, N. A. & Smith, F. A. Evidence for mesothermy in dinosaurs. *Science* **344**, 1268 LP–1272 (2014).
7. McAllister, R. P. et al. Late Jurassic climates, vegetation, and dinosaur distributions. *J. Geol.* **112**, 643–653 (2004).

8. Close, R. A., Benson, R. B. J., Upchurch, P. & Butler, R. J. Controlling for the species-area effect supports constrained long-term Mesozoic terrestrial vertebrate diversification. *Nat. Commun*. **8**, 15381 (2017).
9. Plotnick, R. E, Recurrent hierarchical patterns and the fractal distribution of fossil localities. *Geology*. **45**, 295–298 (2017).
