# Supplementary material for: Ecological niche modelling does not support climatically-driven dinosaur diversity decline before the Cretaceous/Paleogene mass extinction
Source: Nat Commun. 2019 Mar 6;10:1091. doi: 10.1038/s41467-019-08997-2 (PMC6403247; doi:10.1038/s41467-019-08997-2)
Supplement: Supplementary file 3 — Reporting Summary [file 41467_2019_8997_MOESM3_ESM.pdf]

nature research

Corresponding author(s): Alfio Alessandro Chiarenza

Last updated by author(s): Jan 20, 2019

Reporting Summary

Nature Research wishes to improve the reproducibility of the work that we publish. This form provides structure for consistency and transparency in reporting. For further information on Nature Research policies, see Authors & Referees and the Editorial Policy Checklist.

Statistics

For all statistical analyses, confirm that the following items are present in the figure legend, table legend, main text, or Methods section.

n/a	Confirmed	
☐	☒	The exact sample size (*n*) for each experimental group/condition, given as a discrete number and unit of measurement
☒	☐	A statement on whether measurements were taken from distinct samples or whether the same sample was measured repeatedly
☐	☒	The statistical test(s) used AND whether they are one- or two-sided<br>*Only common tests should be described solely by name; describe more complex techniques in the Methods section.*
☐	☒	A description of all covariates tested
☐	☒	A description of any assumptions or corrections, such as tests of normality and adjustment for multiple comparisons
☐	☒	A full description of the statistical parameters including central tendency (e.g. means) or other basic estimates (e.g. regression coefficient) AND variation (e.g. standard deviation) or associated estimates of uncertainty (e.g. confidence intervals)
☐	☒	For null hypothesis testing, the test statistic (e.g. *F*, *t*, *r*) with confidence intervals, effect sizes, degrees of freedom and *P* value noted<br>*Give P values as exact values whenever suitable.*
☒	☐	For Bayesian analysis, information on the choice of priors and Markov chain Monte Carlo settings
☒	☐	For hierarchical and complex designs, identification of the appropriate level for tests and full reporting of outcomes
☐	☒	Estimates of effect sizes (e.g. Cohen's *d*, Pearson's *r*), indicating how they were calculated

Our web collection on statistics for biologists contains articles on many of the points above.

Software and code

Policy information about availability of computer code

Data collection	Occurrences data attached to manuscript and publicly available at: https://paleobiodb.org/#/
Data analysis	Geospatial analyses and ecological modelling performed in ArcGis 10.2.2 (ESRI) and R version 3.4.4 (R Development Core Team, 2017). Custom codes available on FigShare (DOI: 10.6084/m9.figshare.7609229 and DOI: 10.6084/m9.figshare.7609226).

For manuscripts utilizing custom algorithms or software that are central to the research but not yet described in published literature, software must be made available to editors/reviewers. We strongly encourage code deposition in a community repository (e.g. GitHub). See the Nature Research guidelines for submitting code & software for further information.

Data

Policy information about availability of data

All manuscripts must include a data availability statement. This statement should provide the following information, where applicable:

- Accession codes, unique identifiers, or web links for publicly available datasets
- A list of figures that have associated raw data
- A description of any restrictions on data availability

The authors declare that all the data supporting the findings of this study are available within the paper and its Supplementary Information files and at at: http://www.bridge.bris.ac.uk/resources/simulations.

Field-specific reporting

Please select the one below that is the best fit for your research. If you are not sure, read the appropriate sections before making your selection.

☐ Life sciences ☐ Behavioural & social sciences ☒ Ecological, evolutionary & environmental sciences

For a reference copy of the document with all sections, see nature.com/documents/nr-reporting-summary-flat.pdf

Ecological, evolutionary & environmental sciences study design

All studies must disclose on these points even when the disclosure is negative.

Study description	We used ecological niche modelling to reproduce habitat suitability in the last approximately 16 million years of non-avian dinosaurs evolutionary history. We found no evidence in support for a long term dinosaur extinction caused by climatic fluctuactions. Instead, by using geological modelling, we highlight how preservational biases may have played a role on the supposedly declining trend of dinosaur diversity on the lead-up to the end-Cretaceous mass extinction.
Research sample	Fossil occurrences, climatic data from General Circulation Models, Palaeogeographic reconstructions.
Sampling strategy	Downloading and vetting the data from The Palebiology Database (https://paleobiodb.org/).
Data collection	Downloading and vetting the data from The Palebiology Database (https://paleobiodb.org/).
Timing and spatial scale	Instant download - months (>10) of data vetting and preparation. Climatic data from general circulation models took years in the making.
Data exclusions	Data with poor chronostratigraphic, spatial and taxonomic control were excluded from the study.
Reproducibility	Codes and methodologies available present in the literature and available to ensure the best practice particular in ecological modelling.
Randomization	Correlation were tested for climatic variables using Pearson's correlation test (details in the Method section of the paper).
Blinding	Community driven data collection.

Did the study involve field work? ☐ Yes ☒ No

Reporting for specific materials, systems and methods

We require information from authors about some types of materials, experimental systems and methods used in many studies. Here, indicate whether each material, system or method listed is relevant to your study. If you are not sure if a list item applies to your research, read the appropriate section before selecting a response.

Materials & experimental systems

n/a	Involved in the study	
☒	☐	Antibodies
☒	☐	Eukaryotic cell lines
☐	☒	Palaeontology
☒	☐	Animals and other organisms
☒	☐	Human research participants
☒	☐	Clinical data

Methods

n/a	Involved in the study	
☒	☐	ChIP-seq
☒	☐	Flow cytometry
☒	☐	MRI-based neuroimaging

Palaeontology

Specimen provenance	Latest Cretaceous (approximately 83-66 million years ago).
Specimen deposition	All specimen information were accessed via public repository deposited online at The Paleobiology Database (https://paleobiodb.org/).
Dating methods	As reported in The Paleobiology Databse for each entries (Downloading and vetting the data from The Palebiology Database (https://paleobiodb.org/). All occurrences and relative information are reported in Supplementary Table 1.

☒ Tick this box to confirm that the raw and calibrated dates are available in the paper or in Supplementary Information.
